# The *LSP1* rs3817198 T > C polymorphism contributes to increased breast cancer risk: a meta-analysis of twelve studies

**DOI:** 10.18632/oncotarget.11741

**Published:** 2016-08-31

**Authors:** Jianzhou Tang, Hui Li, Jiashun Luo, Hua Mei, Liang Peng, Xiaojie Li

**Affiliations:** ^1^ Department of Biological and Environmental Engineering, Changsha University, Changsha 410003, Hunan, China; ^2^ Department of Microbiology and Immunology, Medical School of Jishou University, Jishou 416000, Hunan, China; ^3^ Institute of Medical Sciences, Medical School of Jishou University, Jishou 416000, Hunan, China; ^4^ Hunan Guangxiu Hospital, Changsha 410002, Hunan, China; ^5^ College of Animal Science and Technology of Hunan Agriculture University, Changsha 410128, Hunan, China

**Keywords:** LSP1, breast cancer, risk, meta-analysis

## Abstract

The association between the *LSP1* rs3817198 T > C polymorphism and breast cancer risk has been widely investigated, but remains controversial. We therefore undertook a comprehensive meta-analysis to provide a high-quality evaluation of this association. A literature search was performed among Pubmed, EMBASE and Chinese National Knowledge Infrastructure (CNKI) databases prior to July 31, 2016, and the strength of the association between the *LSP1* rs3817198 T > C polymorphism and breast cancer risk was assessed based on odds ratio (OR) and 95% confidence interval (95% CI). In total, 12 studies with 50,525 cases and 54,302 controls were included. Pooled risk estimates indicated a significant association between the *LSP1* rs3817198 T > C polymorphism and breast cancer risk. Analysis of cases stratified based on ethnicity suggested that the association was significant in both Caucasian and Asian populations. Stratification based on source of controls revealed an association only in population-based studies. These findings indicate the *LSP1* rs3817198 T > C polymorphism is associated with increased risk of breast cancer, especially in Caucasian and Asian populations. Large, well-designed studies with different ethnicities are still needed to verify our findings.

## INTRODUCTION

In 2012, there were approximately 1.7 million newly diagnosed breast cancer patients and 521,900 deaths, accounting for 25% of all new cancer cases and 15% of all cancer-related death in women [1]. Despite the prevalence and severity of breast cancer, the exact mechanism underlying the initiation and progression of breast cancer is still not fully understood. Breast cancer is caused by the interaction of various environmental and genetic risk factors [2, 3]. Environmental variables, such as reproductive factors, hormonal stimulation, high birth weight, obesity, physical inactivity, and alcohol consumption are well-established breast cancer risk factors [4–6]. Moreover, germline mutations in some highly and moderately penetrant genes, including *BRCA1*, *BRCA2*, *PTEN*, *TP53*, *CHEK2*, *ATM*, *BRIP1* and *PALB2*, are associated with high and moderate risk of breast cancer [7, 8]. However, mutations in these genes only explain 25% of breast cancer risk [9]. Single nucleotide polymorphisms (SNPs) in some genes can alter mRNA and protein expression or protein function, and thereby influence cancer susceptibility. A recent genome wide association study (GWAS) has discovered SNPs in 5 lowly penetrant genes as additional susceptibility factors with high frequency, and validated their strong association with breast cancer [10].

One of these genes, lymphocyte-specific protein 1 (*LSP1*), is located on chromosome 11p15.5. It encodes an F-actin bundling cytoskeletal protein expressed in hematopoietic and endothelial cells [8, 10]. Many polymorphisms in the *LSP1* gene have been identified, and one of the most common polymorphisms, the *LSP1* s3817198 T > C, has been widely studied for its potential association with breast cancer risk. Several publications have reported a significant association of the *LSP1* rs3817198 T > C polymorphism with the risk of breast cancer [11–13]. However, other studies have failed to replicate such an association [14–16]. Chen et al. [17] conducted a meta-analysis in 2010, and concluded that the *LSP1* rs3817198 T > C polymorphism was significantly correlated with breast cancer risk. However, only seven studies were available at that time. Since then, some new case-control studies evaluating the association have emerged [13, 16, 18–20]. Therefore, we performed an updated meta-analysis to provide a more comprehensive and accurate assessment of the association between the *LSP1* rs3817198 T > C polymorphism and breast cancer risk.

## RESULTS

### Study characteristics

**Figure 1:**
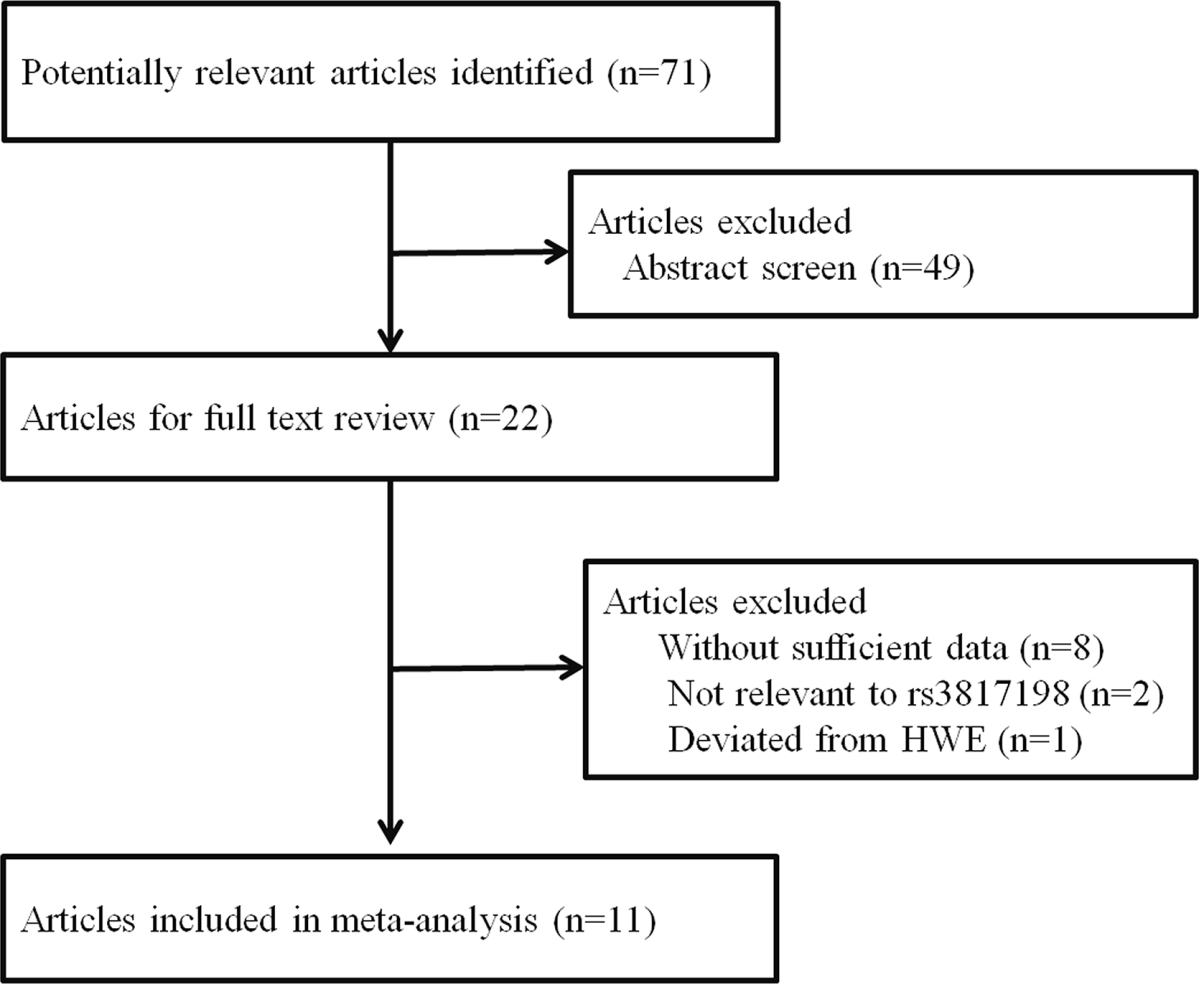
Flowchart of articles included in the meta-analysis

As shown in Figure 1, a total of 71 articles were found from Pubmed, EMBASE, and Chinese National Knowledge Infrastructure (CNKI) databases with the use of specific search terms. Of these, 49 articles were excluded after reviewing the titles and abstracts. The remaining 22 articles were subsequently evaluated for full-text review. Another 11 articles were excluded because they lacked sufficient data, were not relevant to the rs3817198 polymorphism, or not in compliance with Hardy-Weinberg equilibrium (HWE). Finally, 11 articles were eligible for the meta-analysis [11–14, 16, 18–23]. Among them, one article reported the association separately in both Caucasian and African populations, thus we extracted two independent studies from the investigation [11]. In the end, 11 articles with 12 studies, comprising 50,525 cases and 54,302 controls were included in our meta-analysis. As listed in Table 1, 6 studies were conducted in Caucasians, 3 in Asians, 1 in Africans, and 2 in mixed populations. Of the 12 studies, 7 were population-based, 2 were hospital-based, and 3 were nested. The genotype frequency distribution of the *LSP1* rs3817198 T > C polymorphism in controls was in compliance with HWE in all studies. Furthermore, 10 articles were considered high quality (quality score ≥ 9), and only 2 were considered low quality (quality score < 9).

**Table 1:** Characteristics of included studies in this meta-analysis

Surname	Year	Country	Ethnicity	Source of control	*N* (cases/controls)		MAF	HWE	Score
Antoniou	2008	UK	Caucasian	Nested	7811	6607	0.32	0.413	9
Garcia-Closas	2008	USA	Mixed	Nested	22397	26012	0.30	0.398	9
Barnholtz-Sloan	2010	USA	African	PB	742	658	0.17	0.157	14
Barnholtz-Sloan	2010	USA	Caucasian	PB	1228	1117	0.31	0.332	14
Gorodnova	2010	Russia	Caucasian	PB	140	174	0.28	0.856	11
Latif	2010	UK	Caucasian	HB	922	366	0.33	0.938	12
Tamimi	2010	USA	Caucasian	PB	680	737	0.29	0.400	13
Long	2010	China	Asian	PB	6435	3839	0.12	NA	8
Campa	2011	Germany	Mixed	Nested	8292	11558	0.30	0.779	7
Jiang	2011	China	Asian	PB	492	510	0.14	0.078	12
Butt	2012	Sweden	Caucasian	PB	689	1330	0.29	0.579	9
Sueta	2012	Japan	Asian	HB	697	1394	0.15	0.367	11

PB, population based; HB, hospital based; MAF, Minor Allele Frequency; HWE, Hardy-Weinberg equilibrium; NA, not available.

### Meta-analysis results

**Figure 2:**
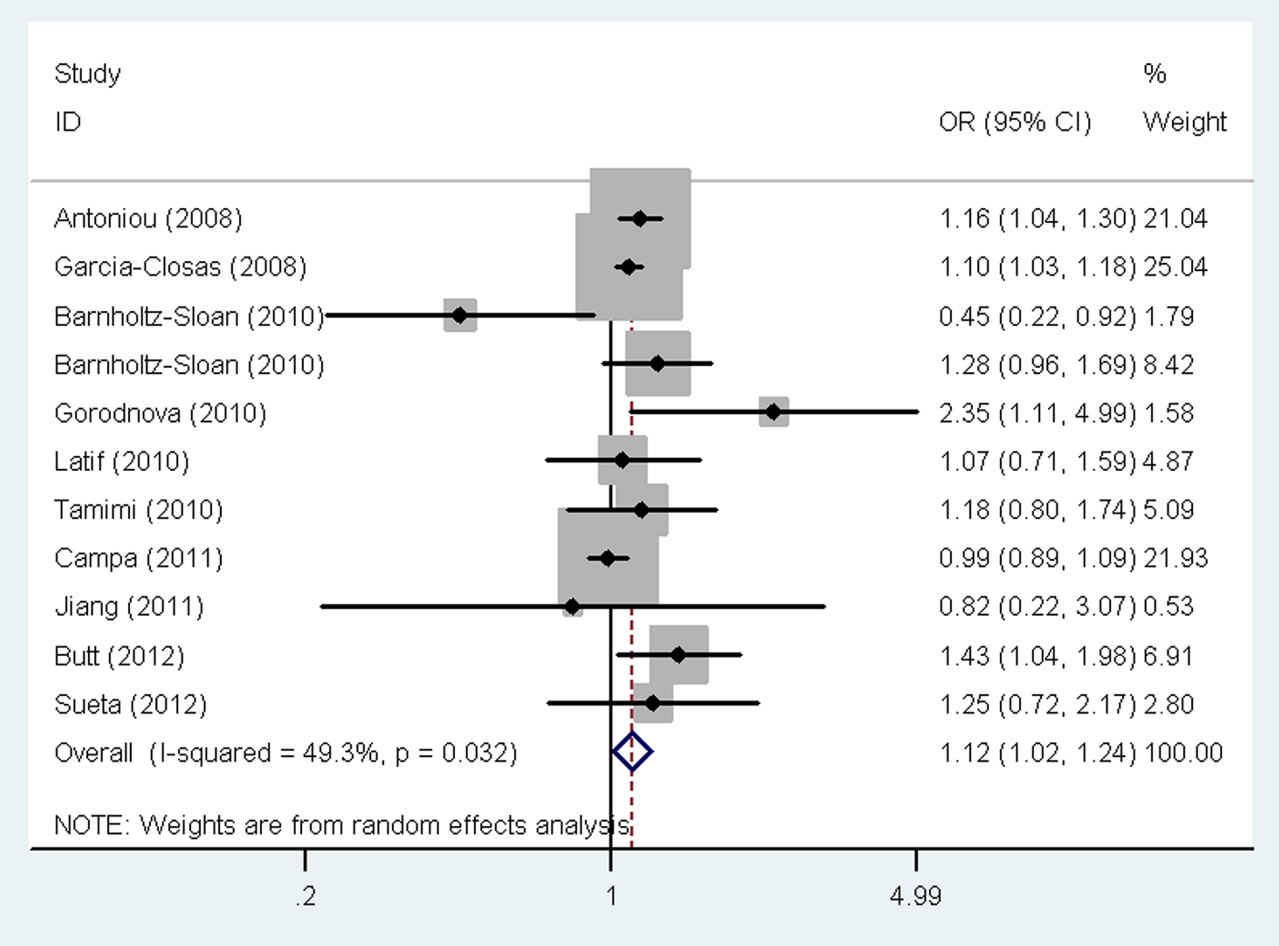
Forest plot of the association between the *LSP1* rs3817198 T > C polymorphism and breast cancer risk under a homozygous model

**Figure 3:**
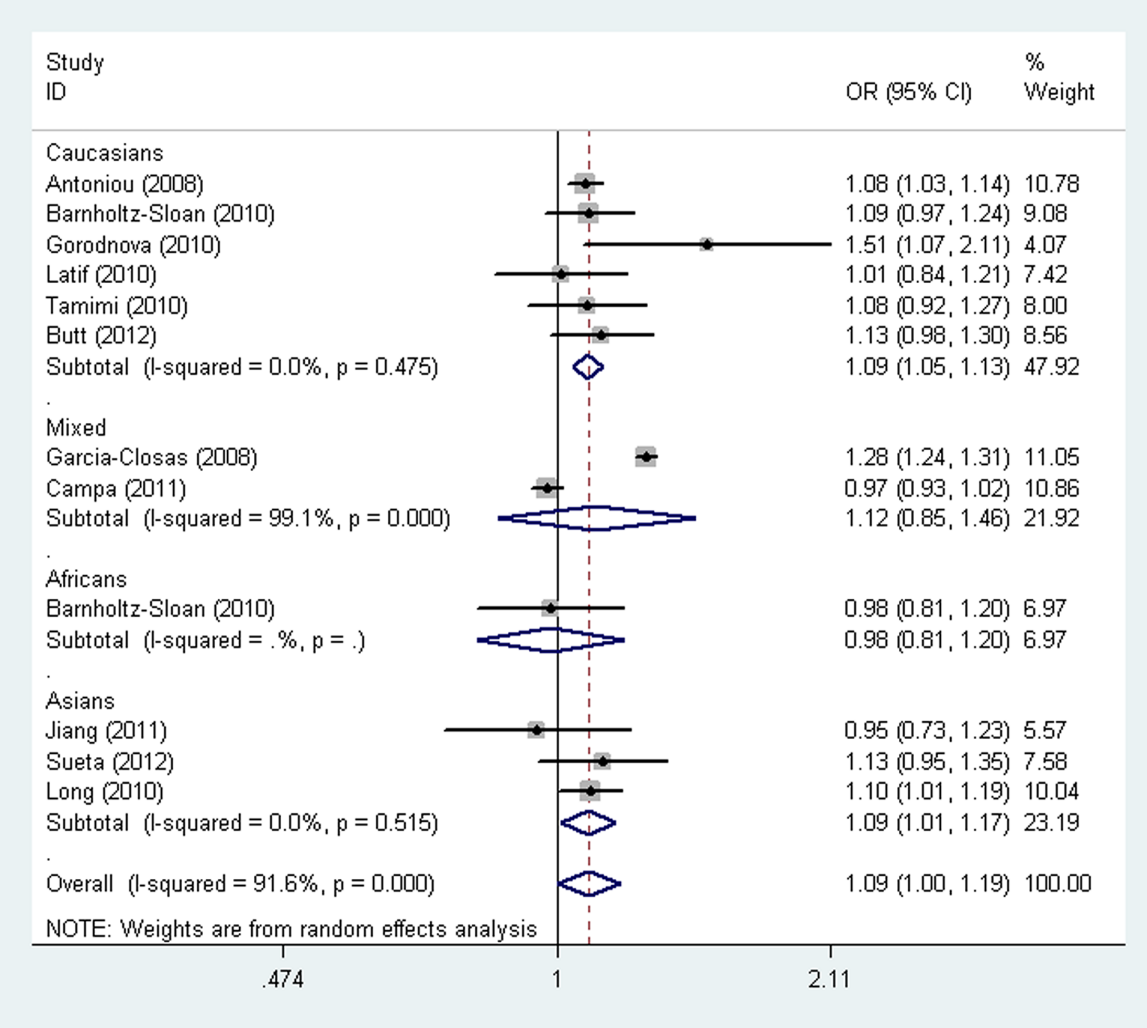
Forest plot of the association between the *LSP1* rs3817198 T > C polymorphism and breast cancer risk stratified by ethnicity under an allele contrast model

**Figure 4:**
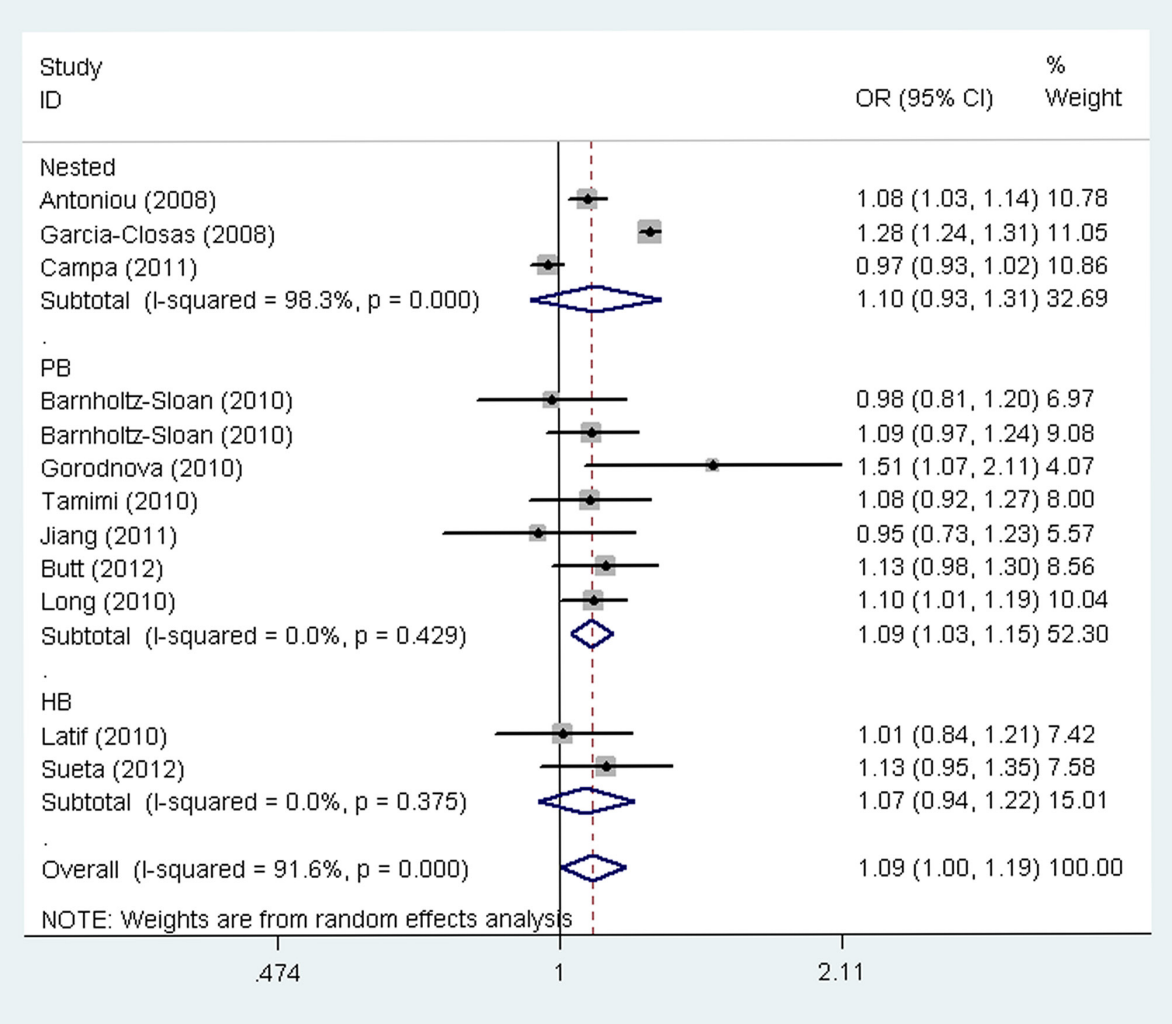
Funnel plot analysis for publication bias by source of control under an allele contrast model

The main results of the meta-analysis for the association between the *LSP1* rs3817198 T > C polymorphism and breast cancer risk are listed in Table 2. Pooled analysis indicated that there was a significant association between the *LSP1* rs3817198 T > C polymorphism and increased breast cancer risk (homozygous model (CC vs. TT): odds ratio (OR) = 1.12, 95% confidence interval (CI) = 1.02–1.24, *P* = 0.021, Figure 2; as well as comparison of allele frequencies (C vs. T): OR = 1.09, 95% CI = 1.00–1.19, *P* = 0.039). Stratified analysis by ethnicity revealed an increased risk of breast cancer associated with rs3817198 T > C in Caucasian populations (homozygous model: OR = 1.21, 95% CI = 1.10–1.32, *P* < 0.001; heterozygous model (TC vs. TT): OR = 1.07, 95% CI = 1.01–1.13, *P* = 0.017; recessive model (CC vs. TC + TT): OR = 1.16, 95% CI = 1.07–1.27, *P* = 0.001; dominant model (TC +CC vs. TT): OR = 1.10, 95% CI = 1.04–1.16, *P* = 0.001; as well as comparison of allele frequencies: OR = 1.09, 95% CI = 1.05–1.13, *P* < 0.001, Figure 3), and also in Asian populations (comparison of allele frequencies model: OR = 1.09, 95% CI = 1.01–1.17, *P* = 0.023, Figure 3). Additionally, in the stratified analysis by source of controls, it was noted that the *LSP1* rs3817198 variant allele (C) was significantly associated with an increased breast cancer risk in population-based studies (comparison of allele frequencies model: OR = 1.09, 95% CI = 1.03–1.15, *P* = 0.001, Figure 4).

**Table 2:** Results of meta-analysis for the association between *LSP1* rs1817198 T > C polymorphism and breast cancer risk

Variables	N (cases/controls)	Homozygous			Heterozygous			Recessive			Dominant			Allele		
		CC vs. TT			TC vs. TT			CC vs. (TC + TT)			(TC +CC) vs. TT			C vs. T		
		OR (95% CI)	*P*^het^	*I*^2^ (%)	OR (95% CI)	*P*^het^	*I*^2^ (%)	OR (95% CI)	*P*^het^	*I*^2^ (%)	OR (95% CI)	*P*^het^	*I*^2^ (%)	OR (95% CI)	*P*^het^	*I*^2^ (%)
All	12(50,525/54302)	**1.12 (1.02-1.24)**	0.032	49.3	1.12 (0.90-1.39)	<0.001	97.6	1.07 (0.91-1.25)	<0.001	82.1	1.13 (0.94-1.36)	<0.001	97.1	**1.09 (1.00-1.19)**	<0.001	91.6
Ethnicity																
Caucasian	6(11470/10331)	**1.21 (1.10-1.32)**	0.408	1.4	**1.07 (1.01-1.13)**	0.812	0.0	**1.16 (1.07-1.27)**	0.436	0.0	**1.10 (1.04-1.16)**	0.686	0.0	**1.09 (1.05-1.13)**	0.475	0.0
Asian	3(7624/5743)	1.17 (0.70-1.95)	0.563	0.0	1.07 (0.90-1.26)	0.324	0.0	1.14 (0.69-1.89)	0.606	0.0	1.07 (0.89-1.28)	0.277	15.4	**1.09 (1.01-1.17)**	0.515	0.0
African	1(742/658)	0.45 (0.22-0.92)	-	-	1.16 (0.92-1.47)	-	-	0.43 (0.22-0.88)	-	-	1.08 (0.86-1.35)	-	-	0.98 (0.81-1.20)	-	-
Mixed	2(30689/37570)	1.05 (0.94-1.17)	0.082	67.0	1.29 (0.70-2.41)	<0.001	99.7	0.90 (0.72-1.13)	<0.001	93.1	1.26 (0.73-2.17)	<0.001	99.6	1.12 (0,85-1.46)	<0.001	99.1
Source of control																
Nested	3(38500/44177)	1.08 (0.99-1.18)	0.076	61.2	1.22 (0.79-1.88)	<0.001	99.5	0.97 (0.79-1.19)	<0.001	93.8	1.20 (0.83-1.74)	<0.001	99.4	1.10 (0.93-1.31)	<0.001	98.3
PB	7(10406/8365)	1.19 (0.88-1.63)	0.036	57.9	1.06 (0.97-1.17)	0.763	0.0	1.16 (0.86-1.56)	0.040	57.1	1.09 (0.99-1.19)	0.644	0.0	**1.09 (1.03-1.15)**	0.429	0.0
HB	2(1619/1760)	1.13 (0.81-1.56)	0.651	0.0	1.07 (0.91-1.25)	0.332	0.0	1.12 (0.82-1.54)	0.756	0.0	1.08 (0.92-1.26)	0.345	0.0	1.07 (0.94-1.22)	0.375	0.0

PB, population based; HB, hospital based.

### Heterogeneity and sensitivity analyses

There were significant heterogeneities detected while evaluating the association between the *LSP1* rs3817198 T > C polymorphism and breast cancer risk under all five genetic models (homozygous model: *P* = 0.032; heterozygous model: *P* < 0.001; recessive model: *P* < 0.001; dominant model: *P* < 0.001; comparison of allele frequencies: *P* < 0.001). Thus, the random-effects model was applied to calculate pooled ORs and 95% CIs. The leave-one-out sensitivity analysis found that no single study had qualitatively altered the pooled ORs, suggesting that our meta-analysis were relatively robust.

### Publication bias

Egger's test was used to assess publication bias in this meta-analysis. No publication bias was found for any of the five models (homozygous model: *P* = 0.637; heterozygous model: *P* = 0.156; recessive model: *P* = 0.191; dominant model: *P* = 0.194; comparison of allele frequencies: *P* = 0.268).

## DISCUSSION

The *LSP1* gene encodes an F-actin bundling protein, which is expressed in lymphocytes, neutrophils, and endothelial cells. LSP1 protein regulates neutrophil motility, adhesion to fibrinogen matrix proteins, and transendothelial migration [24, 25]. Polymorphisms in the *LSP1* gene may lead to alterations in the expression and function of the protein as well as the regulation of downstream signaling pathways, thereby modulating breast cancer susceptibility [7, 8, 26]. The *LSP1* rs3817198 T > C polymorphism has been widely studied for its potential association with the risk of breast cancer; however, the findings were inconclusive. This updated meta-analysis was performed to draw a more precise conclusion about the association, with the addition of recently published studies. In the current meta-analysis, a total of 50,525 cases and 54,302 controls were retrieved to assess the association between the *LSP1* rs3817198 T > C polymorphism and breast cancer risk. We found that an increased risk of breast cancer was observed for the *LSP1* rs3817198 T > C polymorphism under both a homozygous model and a comparison of allele frequencies model. Further stratified analysis showed that this association was notable in Caucasian populations, Asian populations, and in population-based studies. Our results suggest that the *LSP1* rs3817198 T > C polymorphism is a risk factor for breast cancer.

Previously, only one meta-analysis (in 2011) investigated the association between the *LSP1* rs3817198 T > C polymorphism and breast cancer risk [17]. The previous meta-analysis included only 7 studies with 33,920 cases and 35,671 controls and found a significant association between the *LSP1* rs3817198 T > C polymorphism and breast cancer under homozygous model and comparison of allele frequencies model. A number of new studies [13, 16, 18–20] comprising 16,605 cases and 18,631 controls were also included in our current meta-analysis. As a result, the statistical power of our meta-analysis was greatly increased. Consistent with the previous meta-analysis, we observed an increased risk of breast cancer associated with the *LSP1* rs3817198 T > C polymorphism under both a homozygous model and a comparison of allele frequencies model. Stratification analysis in the previous meta-analysis also indicated that the *LSP1* rs3817198 T > C polymorphism was significantly associated with breast cancer in Caucasians under homozygous and recessive models and in mixed ethnicities under a homozygous model [17]. However, our meta-analysis showed that the *LSP1* rs3817198 T > C polymorphism was significantly associated with breast cancer in Caucasians under all five genetic models and we failed to replicate the association for mixed ethnicities. These discrepancies between the two meta-analyses may be accredited to the differences in the sample size and the classification of ethnicities. Chen et al. did not include studies conducted among Asians, possibly leading to bias in their results. Our meta-analysis included 3 studies performed among Asian populations [18–20]. We also first found an increased risk of breast cancer with the *LSP1* rs3817198 T > C polymorphism in an Asian population, although there was a stronger association in the Caucasian population. Moreover, a significantly elevated risk of breast cancer in nested case-control studies was observed by Chen et al. [17] and was not replicated in our meta-analysis. Instead, we found that the polymorphism increased the risk of breast cancer by at least 9% in population-based studies, which may be attributed to our relatively large sample size.

Several limitations to our meta-analysis should be noted. First, in the stratification analysis by ethnicity, the numbers of studies among Asian and Africans were relatively small. Therefore, the statistical power might be not sufficient to assess the relationship. Second, the source of controls was not uniformly defined. Some studies adopted population-based controls or hospital-based controls, while other studies had nested controls. Third, our meta-analysis results were based on unadjusted risk estimates. We did not have sufficient data to conduct a more precise analysis with adjustment for age, obesity, smoking, drinking, menopausal status, environmental factors and lifestyle. Nonetheless, our meta-analysis provides a more comprehensive assessment of the association between the *LSP1* rs3817198 T > C polymorphism and breast cancer risk, and is based on a relatively large sample size. Our results indicate that the *LSP1* rs3817198 T > C polymorphism increases susceptibility to breast cancer, especially in Caucasian and Asian populations.

## MATERIALS AND METHODS

### Identification of eligible relevant studies

To retrieve all eligible articles that assessed the association between the *LSP1* rs3817198 T > C polymorphism and breast cancer risk, we performed a literature search using Pubmed, EMBASE, and Chinese National Knowledge Infrastructure (CNKI) databases prior to July 31, 2016. The search terms that used were as follows: “Lymphocyte-specific protein 1 or LSP1”, “variant or polymorphism” and “cancer or tumor or carcinoma”. Reference lists of relevant studies and review articles were also screened manually for additional eligible articles. Only articles written in English and Chinese were retrieved for further screening.

### Inclusion and exclusion criteria

Eligible studies had to satisfy all of the following criteria: (a) case-control studies in human populations; (b) investigation of the association of the *LSP1* rs3817198 T > C polymorphism with breast cancer risk; (c) sufficient information for estimating the ORs and 95% CIs; and (d) genotype frequency distributions in the control group in compliance with HWE. The exclusion criteria were: (a) not a case-control study; (b) abstracts, reviews, or comments; (c) lacking sufficient data; (d) replicating data. If several studies shared the same or overlapping subjects, only the most recent study or the study with the largest number of participants or most complete data was selected.

### Data extraction

Two authors independently reviewed and extracted the information from the studies and applied the inclusion and exclusion criteria. If any discrepancy was encountered, a consensus was finally reached by consultation and discussion with a third author. The following data were extracted from each eligible study: the surname of the first author, year of publication, country of origin, ethnicity, source of control, number of cases and controls, allele or genotype frequencies of the *LSP1* rs3817198 for cases and controls, evidence of HWE, and quality score (high quality articles with score ≥ 9, low quality articles with score < 9) [27, 28].

### Statistical analysis

The strength of the association between the *LSP1* rs3817198 T > C polymorphism and the risk of breast cancer was assessed by ORs and corresponding 95% CIs under five different genetic models. The models were as follows: homozygous model (CC vs. TT), heterozygous model (TC vs. TT), recessive model (CC vs. TC + TT) and dominant model (TC +CC vs. TT), as well as comparison of allele frequencies (C vs. T). We use the Q-statistic to evaluate between-study heterogeneity. For the *Q* test, a *P* value greater than 0.10 indicated a lack of heterogeneity. In the case of no heterogeneity, the fixed-effects model (Mantel-Haenszel method) was applied [29]. Otherwise, the random-effects model (the DerSimonian and Laird method) was selected [30]. In addition, the *I*^2^ test was also used to quantify the heterogeneity among studies [31]. We also conducted sensitivity analysis to assess the stability of our meta-analysis. In order to do so, we consecutively omitted one study at a time and recalculated OR and 95% CI. Funnel plots and Egger's linear regression test was used to check for publication bias [32]. All statistical analyses were conducted with STATA Software (version 11.0; Stata Corporation, College Station, TX).
